# Retrospective comparison of death or neurodevelopmental outcomes in extremely low birth weight preterm infants following different management options of haemodynamically significant patent ductus arteriosus

**DOI:** 10.1186/s12887-021-02920-9

**Published:** 2021-10-19

**Authors:** Jania Jia-Ying Wu, Krishnamoorthy Niduvaje, Le ye Lee, Zubair Amin

**Affiliations:** 1 Department of Paediatrics, Yong Loo Lin School of Medicine, National University of Singapore, Singapore, Singapore; 2 Department of Neonatology, Khoo Teck Puat-National University Children’s Medical Institute, National University Healthcare System, Singapore, Singapore

**Keywords:** Chronic lung disease, Conservative treatment, Death, Extremely preterm infants, Neurodevelopmental delay

## Abstract

**Background:**

Optimal management of haemodynamically significant patent ductus arteriosus (HsPDA) in premature babies remains controversial. Our aim is to compare death and/or adverse neurodevelopmental outcomes in extremely low birth weight (ELBW) infants with HsPDA who were managed with conservative [C], medical [M] and/or surgical [S] treatment, with secondary aim to examine short-term morbidities among [S] and [C] groups. The study also compared outcomes in very low birth weight (VLBW) infants with HsPDA and non-HsPDA.

**Methods:**

A retrospective study of VLBW preterm infants born before 29 weeks in Singapore from 2007 to 2016 was conducted.

**Results:**

A total of 474 VLBW infants were admitted in NUH from 2007 to 2016. Infants aged between 24 + 0 and 28 + 6 weeks of gestation, weighing ≤1500 g and diagnosed with patent ductus arteriosus (PDA) were included in the study, of which 172 infants (124 HsPDA and 48 non-HsPDA) were analyzed. Among infants with HsPDA, 17 infants were managed with [C], 83 with [M] and 24 with [S]. Mortality was not increased regardless of the presence of HsPDA or treatment received. Infants with non-HsPDA were less likely to have isolated speech delay (*p* < 0.05), but not global developmental delay (GDD). No significant differences in neurodevelopmental outcomes such as hearing loss, cerebral palsy (CP) and speech delay were found. [M + S] infants were at a higher risk of developing chronic lung disease (CLD) (OR 6.83, *p* < 0.05) and short-term growth failure compared to [C] infants. They were significantly shorter and had a smaller head circumference at discharge (*p* < 0.05). [M + S] infants also had elevated creatinine compared to those in group [C] (81.1 ± 24.1 vs 48.3 ± 11.8 umol/L, *p* < 0.000).

**Conclusions:**

Compared to conservative management, infants requiring [M + S] treatment for HsPDA were more likely to have short-term complications such as CLD, elevated creatinine, and poorer growth. Despite a more turbulent postnatal course, death and/or adverse neurodevelopmental outcomes were not worse in infants managed with [M + S].

## What is known

PDA treatment options range from conservative approach to medical and surgical interventions.

Optimal management is controversial.

## What is new

CLD incidence in infant managed medical and/or surgical more common than those managed conservatively.

Elevated creatinine and growth failure are more common in infants managed medically and/or surgically.

Death and adverse neurodevelopment outcomes in ELBWs were comparable at 18 months.

## Background

In term infants, the patent ductus arteriosus (PDA) normally constricts after birth and closes within 24–48 h of life [1]. In preterm infants, the closure is often delayed due to a lower intrinsic tone of the ductus and increased ductus sensitivity to the vasodilating effects of prostaglandin E2 and nitric oxide (NO) [1]. There is an inverse relationship between the rate of ductal closure and the birth weight (BW) and gestational age (GA) [2–5].

In infants with PDA, blood flows from the left to right circulation, i.e., from the aorta into the pulmonary arteries, increasing the blood flow in the pulmonary circulation. This “ductal steal” phenomenon may result in pulmonary over-circulation and diversion of blood from the systemic circulation [6]. Depending on the magnitude of the shunt, infants may be asymptomatic or symptomatic. As there is no universally accepted definition for hemodynamically significant patent ductus arteriosus (HsPDA), HsPDA is often defined based on the patient’s clinical status, clinical signs, 2D echocardiography findings and other objective assessments [7].

Prolonged exposure to PDA is also associated with neonatal mortality and multiple neonatal morbidities such as chronic lung disease (CLD), necrotising enterocolitis (NEC), retinopathy of prematurity (ROP), intraventricular haemorrhage (IVH), periventricular leukomalacia (PVL) and adverse long-term neurodevelopmental outcomes [8]. The management of HsPDA ranges from conservative management with fluid restriction and diuretics, medical treatment with cyclo-oxygenase inhibitors (COX) such as ibuprofen and indomethacin to surgical ligation.

To date, the optimal management of HsPDA remains controversial and is largely guided by professional opinions [9, 10]. The medical treatment adopted in our institution is intravenous ibuprofen or indomethacin. The current treatment regime is ibuprofen 10 mg/kg, 5 mg/kg, 5 mg/kg 24-hourly, which may be given up to two courses. Treatment is initiated preferably after 2 weeks of life to allow for treatment to be avoided in infants whose HsPDA may close spontaneously. Indomethacin was used from 2007 till April 2010 and the dose regimen was 0.2 mg/kg 12-hourly for 3 doses. Diuretic used for the conservative arms mainly consist of frusemide at 0.5 to 1 mg/kg 12-hourly with spironolactone 1 mg/kg 12-hourly. Surgical ligation is considered when the PDA failed to close after conservative or medical treatment and the child remains mechanically ventilated.

The decision to treat HsPDA with medical treatment or surgery has its risks. Treatment with COX inhibitors is associated with renal impairment, intestinal perforation and altered cerebrovascular regulation [11]. Surgical ligation is associated with haemodynamic and respiratory instability [12] and vocal cord palsy in up to 9% of infants with low recovery rates [13].

As the managment option of HsPDA remains controversial, further information such as short-term morbidities and long-term outcomes is important to help guide the phyisicians and parents to determine the most suitable option. The primary objective of this study is to compare the death and/or adverse neurodevelopmental outcomes in early childhood among extremely low birth weight (ELBW) infants with HsPDA who were managed with conservative [C], medical [M] and/or surgical [S] treatment. The secondary objectives are to examine baseline demographics predisposing to [S] treatment and compare neonatal short-term morbidities among the [S] and [C] infants. The study also compared outcomes in very low birth weight (VLBW) infants with HsPDA and non-HsPDA.

## Methods

This is a retrospective study of VLBW infants (BW ≤ 1500 g) born at National University Hospital (NUH), Singapore from 2007 to 2016 and admitted to the neonatal intensive care unit (NICU). Ethics approval was given by the National Health Group Domain Specific Review Board (NHG DSRB ref. 2020/00001).

The inclusion criteria for comparing outcomes of management in HsPDA infants were: (1) between 24 + 0 and 28 + 6 weeks of gestation, (2) VLBW and (3) diagnosed with PDA (either HsPDA or non-HsPDA). Infants with duct dependent cardiac lesions, those with HsPDA who died before the initiation of treatment for PDA closure, those with non-HsPDA who died between day 0–1 (insufficient time to evaluate the presence of HsPDA) and those lost to follow up were excluded. We limited the study to infants < 29 weeks as more mature infants tend to have spontaneous closure of PDA [3, 6, 14].

We defined HsPDA based on combination of echocardiographic and clinical parameters. Echocardiography criteria were: PDA diameter ≥ 1.5 mm, left atrium: aortic root (LA:Ao) ratio ≥ 1.5:1, absent or reverse diastolic flow in the post-ductal descending aorta or organ arteries [4, 6, 15]. Clinical features suggestive of HsPDA include hyperdynamic precordium, bounding pulses, widened pulse pressure and cardiac failure [6, 15, 16]. The assessment is aided by radiological findings of stigmata of pulmonary edema and increasing respiratory support requirements [10]. The diagnosis of HsPDA was first made by the managing neonatologist, who would refer the infant to an independent paediatric cardiologist. The echocardiography was repeated and the decision for treatment was then agreed upon by consensus between treating physicians and parents.

Infants were grouped to have either HsPDA or non-HsPDA. Infants with HsPDA were further divided based on their management: conservative [C], medical [M] or surgical [S] treatment. The primary composite outcome was 1) death and 2) adverse neurodevelopment outcomes up to 5 years. Neurodevelopmental outcomes studied include hearing loss requiring implants/hearing aids, cerebral palsy (CP) and developmental delay. CP was defined if the child had either diplegia, hemiplegia or quadriplegia between 18 and 24 months of age. Developmental delay was assessed from 18 months corrected age and was defined as global developmental delay (GDD) or isolated speech delay upon confirmation by the independent developmental paediatrician and after appropriate psychological testing for age. The secondary outcomes were baseline demographics predisposing to treatment complications and short-term neonatal morbidities. General demographics, antenatal and neonatal data were collected from electronic medical records and include gender, mode of delivery, GA, birth and discharge anthropometric measures. We used the Fenton’s growth chart [17] to derive the Z-score from the GA and respective parameters (birth weight, length and head circumference). Perinatal and immediate neonatal factors, complications associated with treatment and short-term neonatal morbidities were collected. A low 5-min APGAR score was defined as < 7. High Clinical Risk Index for Babies (CRIB II) score was defined as > 12 [18]. Severe respiratory distress syndrome (RDS) was defined as grade 2–4 as per radiological classification of chest X-ray. Grade 1 is mild with fine ground glass shadowing and grade 4 has alveolar shadowing obscuring the cardiac border [19]. Severe IVH was defined as Grade 3 or 4 IVH [20]. Definite NEC was defined as at least Bell’s stage 2 [21] or if complications developed. Severe ROP was defined as threshold ROP stage 3 or above [22]. CLD was defined as infants requiring supplemental O2 or mechanical support with continuous positive airway pressure (CPAP)/ventilator till 36 weeks of post-menstrual age [23]. For this study purpose, mortality was defined as death up to 12 months after initial discharge from the hospital. Treatment specific medical complications like raised serum creatinine and spontaneous intestinal perforation were collected. Surgical complications like vocal cord paralysis post PDA ligation and aspiration pneumonia were also determined. We also calculated the time to surgical intervention and divided them into early (less than 30 days) and late (more than 30 days).

Data were analysed using SPSS version 25 (IBM Corp, Armonk, NY, USA). Continuous normally distributed data were presented as mean with standard deviations unless stated otherwise and analysed using Student *t*-test with conservative arm as the comparator. Categorical data were presented as frequency and analysed using Chi Square test with 2 × 2 tables. Odds ratio (OR) was determined by comparing the conservative and treatment arm. Multinomial logistic regression was used for multivariate analysis. Further analysis was conducted for the complications related to surgical ligation. A confidence interval of 95% is provided and a *P*-value of < 0.05 is considered significant.

## Results

**Fig. 1:**
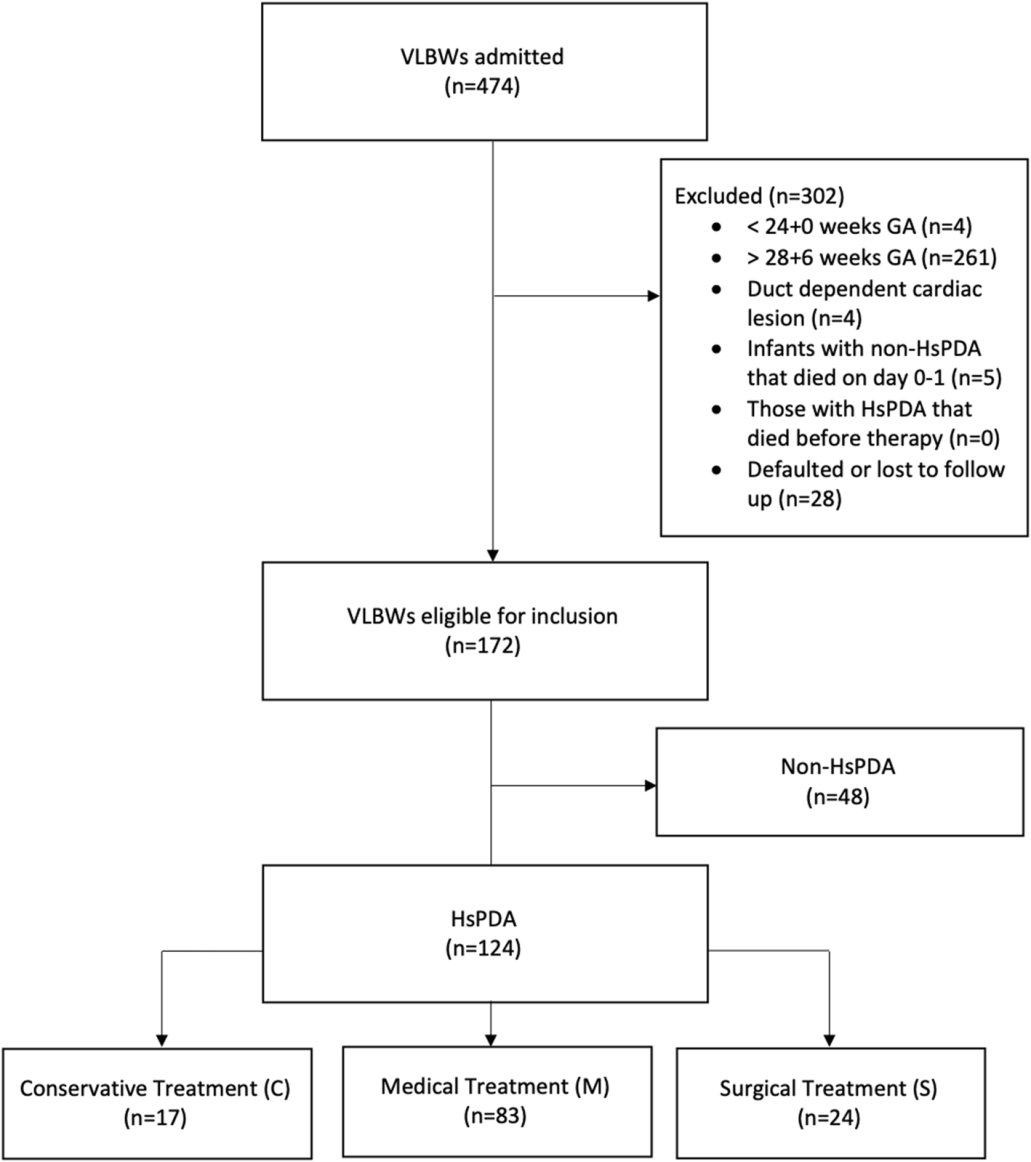
Consort diagram

A total of 474 VLBW infants were admitted in NUH from 2007 to 2016; of which 172 infants (124 HsPDA and 48 non-HsPDA) met study criteria (Fig. 1) and were included in the study for analysis. Of the 474 infants, 438 infants (92.4%) survived to discharge. The yearly incidence of HsPDA ranged from 31.3 to 67.3%. The mean GA was 26.2 ± 1.3 weeks and the mean BW was 890 ± 191 g. 77.4% of infants were singleton pregnancies, 21.8% of infants were twins and 0.8% of infants were triplets. The studied infants were not different from those who defaulted or were lost (*n* = 28, 5.9%) to follow up.

Among the infants with HsPDA, 17 (13.7%) infants were managed conservatively, 83 (66.9%) infants were managed medically and 24 (19.4%) infants were managed with surgical ligation after failure of medical management. Of the 24 infants, 13 underwent surgical treatment before 30 days of age (early) and 11 underwent after 30 days of age (late). Of those managed conservatively, HsPDA was closed in all cases spontaneously before discharge. Of those managed medically alone, all had closure prior to discharge and did not require surgery. All infants with HsPDA that failed to close after medical treatment were managed with surgical ligation.

Of note, there were 13 deaths after treatment was initiated (Table 3); of which 12 infants were treated medically and 1 infant was treated with surgical ligation. Six deaths were due to septicemia, 3 were due to NEC, 2 were due to CLD, 1 was due to extreme prematurity and 1 was due to unrelated causes (age of death: 2Y8M); the last infant was excluded from the analysis as death happened more than 12 months after discharge. None of the deaths was directly due to the presence of PDA or treatment of the PDA (i.e., surgery related).

Logistic regression with multivariate analysis was performed to identify treatment received as a risk factor for death and/or adverse neurodevelopmental outcomes, adjusted for GA, BW z-score, BW, CRIB score more than 12, severity of RDS, development of NEC, sepsis, CLD, weight at discharge z-score, discharge GA and length of stay. Both the length of stay and gestational age at discharge were significant on multivariate analysis.

We analysed outcomes between infants with HsPDA and non-HsPDA. Among infants with HsPDA, we further conducted a main group analysis which compared the outcomes between infants managed conservatively [C] and infants who received either medical and/or surgical treatment [M + S]. Subgroup analysis was performed for baseline demographics characteristics with significant findings on main group analysis (C vs [M + S]) and treatment complications between infants managed [C] and surgically [S].

### General analysis of infants with HsPDA vs non-HsPDA

Table 1 presents the baseline demographics, short-term morbidities and neurodevelopmental outcomes between infants with HsPDA and non-HsPDA. Infants without HsPDA were of significantly older GA, heavier at birth and were less likely to have CRIB II score > 12. At discharge, they were also significantly heavier, taller and had a larger head circumference. Being more mature and heavier, they were also less likely to have severe RDS, requiring less doses of surfactant therapy, and had lower rates of sepsis, CLD and threshold ROP. Non-HsPDA infants were less likely to develop isolated speech delay, but not GDD or death.

**Table 1:** Perinatal characteristics, short-term morbidities and neurodevelopmental outcomes of infants classified by presence of HsPDA

	Non-HsPDA ***n*** = 48	HsPDA ***n*** = 124	
	n (%)/mean ± SD	n (%)/mean ± SD	*P* value
GA (weeks)	27.3 ± 0.8	26.2 ± 1.3	**0.000**
BW (g)	1009.8 ± 212.1	889.6 ± 190.6	**0.000**
APGAR (5 min) < 7	10 (21.3)	14 (11.9)	0.12
CRIB II > 12	5 (10.6)	33 (26.8)	**0.023**
Severe RDS Grade 2–4	11 (22.9)	85 (68.5)	**0.000**
Surfactant Therapy > 2 doses	1 (2.1)	16 (12.9)	**0.033**
Sepsis	6 (12.5)	38 (30.6)	**0.014**
Severe IVH Grade 3–4	5 (10.4)	25 (20.2)	0.13
PVL	1 (2.1)	4 (3.2)	0.69
NEC or NEC with complication/surgery	0 (0.0)	8 (6.5)	0.07
Threshold ROP Stage 3–4	1 (2.4)	24 (21.1)	**0.005**
CLD^a^	34 (81.0)	71 (62.8)	**0.032**
Weight at Discharge z-score	−1.8 ± 1.1	−2.8 ± 1.2	**0.000**
Length at Discharge z-score	−2.1 ± 1.2	−3.6 ± 1.4	**0.000**
Head circumference at Discharge z-score	−0.8 ± 1.5	−2.0 ± 1.0	**0.000**
Death or GDD	37 (77.1)	89 (72.4)	0.53
CP	1 (2.4)	10 (9.0)	0.16
Abnormal hearing test at discharge	1 (2.4)	8 (7.1)	0.26
Hearing loss requiring hearing aids or implants	0 (0.0)	7 (6.3)	0.10
Isolated speech delay	2 (4.8)	19 (17.1)	**0.047**

^a^We assumed the worst case scenario that infants who died prior to 36 weeks developed CLD

### Main group analysis: analysis of infants managed with conservative [C] vs treatment [M + S]

#### Baseline demographics and short-term morbidities

Table 2 presents the baseline demographics and short-term morbidities between infants managed [C] and [M + S]. Analysis of the antenatal and perinatal characteristics revealed that group [M + S] infants were of higher acuity at birth as measured by CRIB II (*p* = 0.046) and had more severe RDS (*p* = 0.04). More than 29% of infants in group [M + S] had a CRIB II score exceeding 12 compared to only 6.3% in those in group [C] (*p* = 0.046) and had more severe RDS (*p* = 0.04). The mean days to surgical ligation was 33.4 (SD 9.4) days. The incidence of CLD in the group [M + S] infants was statistically significant (*p* = 0.005). The OR of group [M + S] infants developing CLD was also significant (OR 6.83 (95% CI 1.49–31.3)) compared to group [C] infants. At discharge, group [M + S] infants were significantly shorter (*p* = 0.017) and had a smaller head circumference (*p* = 0.039) than group [C] infants. However, the weight at discharge z-score between the group [M + S] infants and [C] infants was not statistically significant (*p* = 0.17).

**Table 2:** Perinatal characteristics and short-term morbidities of infants classified by treatment

	Conservative ***n*** = 17	Main group analysis		Subgroup analysis	
		Medical and/or surgical ***n*** = 107		Surgical ***n*** = 24	
	n (%)/mean ± SD	n (%)/mean ± SD	*P* value	n (%)/mean ± SD	*P* value
Perinatal Characteristics					
Gender					
Male	8 (47.1)	61 (57.0)	0.44	12 (50.0)	0.85
Mode of Delivery					
LSCS	8 (47.1)	51 (47.7)	0.96	8 (33.3)	0.38
GA (weeks)	26.5 ± 1.2	26.2 ± 1.3	0.28	25.5 ± 1.0	**0.004**
BW (g)	914.4 ± 160.5	885.6 ± 195.3	0.57	781.8 ± 136.5	**0.007**
Z-score BW	0.05 ± 0.7	−0.01 ± 0.8	0.77	−0.1 ± 0.8	0.40
Z-score birth length	0.04 ± 1.0	− 0.3 ± 1.1	0.19	−0.4 ± 1.1	0.21
Z-score birth head circumference	0.4 ± 0.8	0.08 ± 1.0	0.24	−0.1 ± 0.8	0.07
APGAR (5 min) < 7	2 (13.3)	12 (11.7)	0.85	3 (12.5)	0.97
CRIB II > 12	1 (6.3)	32 (29.9)	**0.046**	11 (45.8)	**0.007**
Perinatal Outcomes					
Severe RDS Grade 2–4	8 (47.1)	77 (72.0)	**0.04**	16 (66.7)	0.21
Surfactant Therapy > 2 doses	2 (11.8)	14 (13.1)	0.88	5 (20.8)	0.43
Sepsis	7 (41.2)	31 (29.0)	0.31	9 (37.5)	0.35
Severe IVH Grade 3–4	0 (0.0)	9 (8.4)	0.21	9 (37.5)	0.06
PVL	0 (0.0)	4 (3.7)	0.42	0 (0.0)	0.36
NEC or NEC with complication/surgery	0 (0.0)	8 (7.5)	0.24	2 (8.3)	0.50
Threshold ROP Stage 3–4	3 (17.6)	21 (21.6)	0.71	9 (37.5)	0.08
CLD^a^	2 (11.8)	51 (47.7)	**0.005**	18 (75.0)	**0.000**
Complications of treatment					
Pulmonary haemorrhage	1 (6.3)	19 (17.8)	0.25	5 (20.8)	0.21
Oliguria (< 1 ml/kg/h)	0 (0.0)	5 (4.7)	0.38	3 (12.5)	0.14
Acute kidney injury	0 (0.0)	4 (3.7)	0.43	2 (8.3)	0.24
No. of courses of medical therapy	0.0 ± 0.0	1.4 ± 0.5	0.45	2.0 ± 0.5	0.06
Highest creatinine	48.3 ± 11.8	81.1 ± 24.1	**0.000**	85.7 ± 32.6	**0.004**
Intestinal perforation	0 (0.0)	5 (4.7)	0.36	1 (4.2)	0.39
Vocal cord paralysis	0 (0.0)	6 (5.6)	0.32	6 (25.0)	**0.026**
Aspiration pneumonia	0 (0.0)	1 (0.9)	0.69	1 (4.2)	0.39
Discharge Parameters of Survivors					
Length of Stay of survivors (days)	87.1 ± 29.3	100.0 ± 38.5	0.19	126.7 ± 39.2	**0.001**
Discharge GA (weeks)	38.9 ± 3.7	40.5 ± 4.9	0.20	43.6 ± 5.6	**0.004**
Weight at Discharge z-score	−2.4 ± 1.0	−2.8 ± 1.2	0.17	−3.4 ± 1.0	**0.003**
Length at Discharge z-score	−2.7 ± 1.1	−3.8 ± 1.4	**0.017**	−4.3 ± 1.1	**0.001**
Head circumference at Discharge z-score	−1.4 ± 0.8	−2.1 ± 1.0	**0.039**	−2.4 ± 1.2	**0.026**

^a^We assumed the worst case scenario that infants who died prior to 36 weeks developed CLD

#### Treatment specific complications

In the analysis of treatment specific complications (Table 2), the only statistically significant finding was the highest measured creatinine level. Highest creatinine was recorded during 2–4 weeks of life in group [C] infants, and up to 72 h from the last dose of COX inhibitors in group [M + S] infants. Of note, one infant in group [C] had an out-of-trend creatinine level of 256 μmol/L due to underlying kidney disease. This infant was excluded from this analysis. The highest measured creatinine was significantly higher in group [M + S] than in group [C] (*p* = 0.000).

#### Death and neurodevelopment outcomes

Table 3 presents death and neurodevelopmental outcomes. The primary composite outcome of death and/or neurodevelopmental delay was not significantly higher in group [M + S] infants as compared to group [C] infants (OR 3.35 (95% CI 0.72–15.5, *p* = 0.105)).

**Table 3:** Primary outcomes death and neurodevelopmental morbidities

	Conservative *n* = 17	Main group analysis	
		Medical and/or surgical *n* = 107	
	n (%)/mean ± SD	n (%)/mean ± SD	*P* value
Death	0 (0.0)	12 (11.2)	0.15
Global developmental delay (GDD)	2 (11.8)	20 (21.3)	0.37
Death or GDD	2 (11.8)	32 (30.2)	0.12
CP	1 (5.9)	9 (9.6)	0.63
Abnormal hearing test at Discharge	1 (5.9)	7 (7.4)	0.83
Hearing loss requiring hearing aids or implants	1 (5.9)	6 (6.4)	0.94
Isolated speech delay	3 (17.6)	16 (17.0)	0.95

### Subgroup analysis: analysis of infants managed with conservative [C] vs surgical [S]

#### Baseline demographics and short-term morbidities

Table 2 presents the baseline demographics and short-term morbidities between infants managed with [C] and [S] treatment. Infants in group [S] were of higher acuity at birth (*p* = 0.007) as measured by CRIB II, but presence of severe RDS was not statistically significant (*p* = 0.21). The incidence of CLD in group [S] was higher than [C] group (*p* = 0.000). Group [S] infants were more likely than group [C] infants to develop CLD. The OR of group [S] infants developing CLD was 22.50 (95% CI 3.95–128.3, *p* = 0.000) when compared to group [C] infants. This is especially so for those who were operated after 30 days of life. There were notable differences in the birth and discharge anthropometric data between the treatment groups. Group [S] infants were significantly younger by 1 week (*p* = 0.004) and lighter by 130 g (*p* = 0.007) as compared to group [C] infants. At discharge, group [S] infants continued to have poorer growth with lower z-scores in length (*p* = 0.001) and head circumference (*p* = 0.026) as compared to group [C].

#### Treatment specific complications

The highest measured creatinine was significantly higher in group [S] (*p* = 0.004) infants than in group [C] infants. The incidence of vocal cord palsy was 25% in group [S] (*p* = 0.026). The OR of developing vocal cord palsy for group [S] as compared to group [C] was 1.94 (95% CI 1.41–2.68, *p* = 0.026) (Table 2).

## Discussion

We examined a cohort of Asian ELBWs with HsPDA. Studies from National Institute of Child Health and Human Development (NICHD) showed that Asian-American are at higher risk of having HsPDA [24]. A comparison between Japan and Canadian neonatal units showed that there is a difference in the composite outcome of morbidity and mortality among VLBW, where the Japanese population (46%) had a lower composite outcome than Canadian population (55%) among infants with PDA (OR 0.70 95% CI 0.62–0.80, *p* < 0.01) [25]. Our study is one of the first to examine the immediate and neurodevelopmental outcomes of following various treatment strategies of HsPDA in the Asian population.

We found that both medical and/or surgical treatment of HsPDA in ELBW are associated with several worse short-term outcomes. ELBW infants who had medical and/or surgical treatment had more CLD incidence, and poorer growth at discharge. We found that the infants in group [M + S] and [S] were sicker at birth compared to infants in group [C] with a higher CRIB II score > 12, predicting a greater risk of neonatal mortality among this group of infants [18]. Base excess, a constituent of the CRIB II score, is an independent predictor of medical PDA closure response [26, 27]. Consequently, it may be challenging to manage the infant with worse base excess conservatively, resulting in the infant receiving either COX inhibitors or surgical ligation. Infants in group [M + S] were more likely to have severe RDS than group [C], in which mechanical ventilation is commonly needed [28]. Mechanical ventilation has been identified to be an independent risk factor for failed medical PDA closure [29], and it is possible that severe RDS is in fact part of the causal pathway of HsPDA requiring medical or surgical treatment. This is also evident in those infants who were operated after 30 days of life as they had longer period of ventilation. Infants in group [S] were also more likely to have vocal cord paralysis than group [C], which is a known complication of surgical ligation [13]. Although [M + S] infants were sicker, had more severe RDS and a larger proportion with higher CRIB II scores, death and/or adverse neurodevelopmental outcomes were not increased in early childhood in the this group. However, a type-II error cannot be excluded as the number of conservatively treated infants was small. At discharge, the group [M + S] infants were smaller in size compared to group [C] infants; where the z-score of length and head circumference were significantly lower. For the cohort, the standardised z-score decreased for all 3 parameters from birth till discharge. The difference between the length and head circumference z-score was more marked between the [M + S] infants and those in [C]. The weight z-score in [C] infants may be also adversely affected by the use of diuretics which leads to a lower weight. Subgroup analysis showed that the group [S] infants were significantly shorter and had smaller head circumference than the group [C] infants at discharge.

The effects of medical or surgical treatment of PDA on neurodevelopmental outcomes has become a topic of increasing interest. Yet, there has been no well-established relationship between the two. Our results found no significant differences in neurodevelopmental outcomes at 18 months of corrected age between infants managed with [M + S] compared to [C]. Furthermore, multivariate analysis found no statistical significance of treatment type received and death and/or adverse neurodevelopmental outcomes except for the duration of hospitalization and the gestational age at discharge. Our findings are consistent with Chorne et al. [30] who found no relationship between surgical ligation and neurodevelopmental outcomes. In contrast, two studies have determined surgical ligation as a higher risk factor for neurodevelopmental impairment as compared to medical treatment [31, 32]. A study reported recently by Janz-Robinson et al. [33] found treatment with both medical and/or surgical treatment to be associated with neurodevelopmental impairment. The discrepancy in results among different studies highlights the gap in the literature and emphasizes the urgent need for large randomized control trials (RCT) to further evaluate the neurodevelopmental outcomes following treatment of HsPDA.

The association between medical treatment and CLD is also not well defined. Our findings suggest that there is a higher incidence and OR of group [M + S] infants developing CLD than group [C] infants, assuming that infants who died prior to 36 weeks had CLD. Prolonged exposure to PDA has been associated with CLD [34, 35]. Our study supports others [36] who found that the adjusted odds of having CLD for ELBW infants with persistent HsPDA is > 3 times that of ELBW infants whose PDAs were successfully closed medically. This is in contrast to the recent PDA-Tolerate study [37] where the authors found no difference in CLD or death between the groups. However, in that study, up to 48% of those in the conservative arm required rescue treatment before discharge. In the medical treatment arm, up to 32% of the infants who received early treatment had treatment failure and subsequently required surgical ligation prior to discharge. As such, these infants may dilute the effects of treatment in the primary outcomes. The discrepancy among studies serves as a call for bigger prospective studies like the BeneDuctus Trial [38], which is currently ongoing, in order to validate the true association between HsPDA exposure, management of HsPDA and CLD.

In our study, we were not able to compare with gestation stratified infants below 24 weeks requiring conservative treatment. This lends further support to earlier studies that found the relationship of the rate of ductal closure with GA and BW [2, 3, 39] to be inversely proportional. Similarly, in another Asian cohort of VLBW infants, a positive correlation between decreasing GA and lower survival was found and the incidence of major neonatal morbidities such as CLD, sepsis, severe ROP and severe IVH were higher with decreasing gestation, although NEC did not show a similar trend [40].

### Limitations and strengths

This study has several limitations due to its retrospective nature. Firstly, there are no definite criteria for the diagnosis of HsPDA as bedside measurement of the PDA can be subjective and the decision for treatment is not standardised due to a lack of universal guidelines. However, as a small unit, we were able to minimise care variations through repeated and consensus discussions. Secondly, there were variations of treatment practices over the years with increasing trend towards conservative treatment. It has only been in the recent years that the trend of PDA treatment has shifted towards a less aggressive approach [24]. As such, majority of the infants with HsPDA in our cohort from 2007 to 2016 were treated either medically or surgically. In addition, it is known from the natural history of PDA that more premature and lower BW infants are less likely to have spontaneous closure [2, 3] and hence there is a lower threshold to treat these infants. It was challenging to find infants managed conservatively, particularly those who were 24 and 25 weeks GA, as we had originally aimed to perform a GA stratified analysis but this was not possible due to the lack of adequate number of infants managed conservatively in this gestation. Early CPAP has also been a standard of care for our unit. More recent approaches and therapy such as INtubation SURfactant Extubation (INSURE) [41] and delayed cord clamping [42, 43] were not practiced during the study period. Thirdly, as surgical ligation was only considered when the PDA failed to close after medical or conservative treatment, it was often performed in potentially sicker infants which can result in more surgically-related side effects. However, none of the infants died as a direct consequence of the surgical intervention or its complications like aspiration or vocal cord palsy. Because it is a retrospective study, we can only establish correlations but not determine the causation between the treatment type and the outcomes. Lastly, this is a single centre study with relatively small sample size, thus a type II error cannot be excluded. A strength of our study is the protocol-based treatment. This reduces the variability in the treatment regimen and improve the quality of our results. As it is a single centre, we have removed the variability between cardiac echocardiographic assessment of the HsPDA and the surgical competency of the surgeon.

## Conclusions

We showed in our cohort of Asian ELBW infants requiring treatment for HsPDA, especially delayed surgical treatment, had more neonatal complications of CLD, elevated creatinine and poorer growth. However, despite a more turbulent postnatal course, they did not have higher incidence of death or adverse neurodevelopmental outcomes at 18-months corrected age.

## Data Availability

Data are confidential. Deidentified data can be shared from the corresponding author on reasonable request.

## Abbreviations

BW: Birth weight
CP: Cerebral palsy
CRIB II: Clinical Risk Index for Babies
CLD: Chronic lung disease
CPAP: Continuous positive airway pressure
C: Conservative treatment
COX: Cyclo-oxygenase inhibitors
ELBW: Extremely low birth weight
GA: Gestational age
HsPDA: Hemodynamically significant patent ductus arteriosus
IVH: Intraventricular haemorrhage
LA:Ao: Left atrium: aortic root
M: Medical treatment
NICHD: National Institute of Child Health and Human Development
NUH: National University Hospital
NEC: Necrotising enterocolitis
NICU: Neonatal intensive care unit
NO: Nitric oxide
PDA: Patent ductus arteriosus
OR: Odds ratio
PVL: Periventricular leukomalacia
RCT: Randomized control trials
RDS: Respiratory distress syndrome
ROP: Retinopathy of prematurity
S: Surgical treatment
VLBW: Very low birth weight
